# Potential Protective and Therapeutic Roles of the Nrf2 Pathway in Ocular Diseases: An Update

**DOI:** 10.1155/2020/9410952

**Published:** 2020-03-23

**Authors:** Ming-xuan Wang, Jing Zhao, Hong Zhang, Ke Li, Ling-zhi Niu, Yuan-ping Wang, Ya-juan Zheng

**Affiliations:** ^1^Department of Ophthalmology, The Second Hospital of Jilin University, Changchun, Jilin, China; ^2^Department of Ophthalmology, China-Japan Union Hospital, Jilin University, Changchun, Jilin, China

## Abstract

Nuclear factor- (erythroid-derived 2-) like 2 (Nrf2) is a regulator of many processes of life, and it plays an important role in antioxidant, anti-inflammatory, and antifibrotic responses and in cancer. This review is focused on the potential mechanism of Nrf2 in the occurrence and development of ocular diseases. Also, several Nrf2 inducers, including noncoding RNAs and exogenous compounds, which control the expression of Nrf2 through different pathways, are discussed in ocular disease models and ocular cells, protecting them from dysfunctional changes. Therefore, Nrf2 might be a potential target of protecting ocular cells from various stresses and preventing ocular diseases.

## 1. Introduction

**Figure 1:**
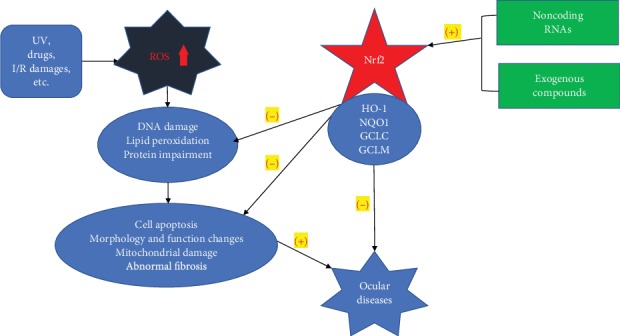
The effects of oxidative stress and the potential protective role of Nrf2 activation.

Oxidative stress (OS) usually comes after an imbalance between reactive oxygen species (ROS) production and elimination as a result of biological system defense mechanisms. In return, OS increases the production of ROS, which creates a vicious cycle. Damages caused by ROS are aimed at deoxyribonucleic acids (DNA), proteins, and lipids and have been observed and studied in corneal diseases [1], cataract [2], retinopathies [3], glaucoma [4], etc. One of the most inspiring discoveries about OS in recent decades has been the elucidation of nuclear factor- (erythroid-derived 2-) like 2 (Nrf2) signaling pathways that regulate OS responses (Figure 1).

Nrf2 is a key regulator of protective antioxidant and anti-inflammatory responses that regulates the expression of hundreds of genes, including not only genes encoding antioxidant enzymes but also a series of genes involved in various processes, including inflammatory responses, cancer occurrence and metastasis, and tissue remodeling and fibrosis [5]. Due to its antioxidative capacity, Nrf2 has been found to mechanistically participate in various systemic diseases, including respiratory diseases [6], cardiovascular and cerebrovascular diseases [7], degenerative diseases, tumors [8], and especially ocular diseases. The Nrf2 signaling system, together with its regulatory molecules and interacting proteins, carries out critical antioxidant and anti-inflammatory functions in cells. Under normal conditions, Nrf2 is sequestered in the cytoplasm, where it mediates proteasomal degradation by binding Kelch-like erythroid cell-derived protein with CNC homology-associated protein 1 (Keap1) to form a complex. Once cellular OS occurs, especially due to exposure to electrophiles including superoxide anion (O_2_^−^), hydrogen peroxide (H_2_O_2_), hydroxyl radical (-OH), and ROS, Keap1 undergoes conformational changes that allow Nrf2 to be transported to the nucleus, where it binds antioxidant response element (ARE) regions. Afterwards, Keap1 initiates transcription of antioxidant and phase II detoxification enzymes, such as NAD(P)H : quinone oxidoreductase 1 (NQO1), heme oxygenase-1 (HO-1), *γ*-glutamyl cysteine ligase catalytic subunit (GCLC), glutathione-S-transferase (GST), glutathione peroxidase (GPX), catalase (CAT), superoxide dismutase (SOD), and thioredoxin UDP-glucuronosyltransferase [9–12]. Alternatively, Nrf2 may be dissociated from the cytoplasmic Nrf2-Keap1-Cul3 complex by p62 (a marker associated with cell autophagy) [13]. Another mechanism is mediated by glycogen synthase kinase 3 (GSK-3) and the E3 ligase adaptor *β*-TrCP [14]. Under normal conditions, GSK-3*α* and *β* remain inactive. However, without receptor signaling, active GSK-3 phosphorylates Nrf2 in its Neh6 domain [15].

Some compounds, especially exogenous compounds including polyphenols, flavonoids, terpenoids, and noncoding ribonucleic acids (RNAs), were reported to be Nrf2 activators or inducers. These compounds may play critical roles in protecting ocular cells against oxidative damage, inflammation, and fibrosis.

## 2. Oxidative Stress and Nrf2 in Ocular Diseases

The eye is an organ subject to constant physical and chemical oxidation. Visible light, ultraviolet (UV) light, ionizing radiation, smog, fine particles in the atmosphere, and other types of pollutants can affect the cornea, lens, and the retina in particular. Correspondingly, OS is associated with many eye diseases [16].

### 2.1. Ocular Surface and Corneal Diseases

Due to its structure and function, the ocular surface and especially the cornea are constantly exposed to high oxygen tension, chemical burns, UV radiation (especially UVB), pathogenic microorganisms, or even urban air pollution [17, 18], which are the source of ROS and OS. The cornea is particularly susceptible to OS due to an imbalance between ROS and cellular antioxidant capacity. Increasing evidence shows that oxidative balance and mitochondrial function are abnormally altered under disease conditions. In addition, oxidative markers such as malondialdehyde (MDA), 4-hydroxynonenal (4-HNE), and nitrotyrosine showed significant changes [19–21]. Nrf2-mediated defense systems are aimed at upregulating the expression of antioxidant proteins and play a key role in protecting cells. Hayashi et al. found that the corneal epithelial wound healing time was much longer in Nrf2 knockout (KO) mice than in the wild-type (WT) mice and that Nrf2 contributed to the healing by accelerating cell migration [22]. Li et al. found that edaravone protected corneal epithelial cells against OS and apoptosis by activating Nrf2 [23]. Mutations in SLC4A11 can cause an increase in the generation of ROS and mitochondrial dysfunction due to oxidative stress [24]. Further study showed that the participation of antioxidative stress in corneal cells and SLC4A11 is necessary for Nrf2-mediated antioxidant gene expression [25].

#### 2.1.1. Keratoconus (KC)

KC is a common degenerative disease of corneal dilatation that usually occurs in adolescence or early adulthood, and it is characterized by a progressive thinning and dilatation of the cornea on both sides, which can appear as a conical protrusion, accompanied by thinning of the central corneal stroma and changes in structural integrity, leading to irregular astigmatism, myopia, and, in severe cases, progressive blurred vision [26, 27]. Visual impairment in some patients with KC can be alleviated with spectacles, specialized contact lenses, or riboflavin-UVA-induced collagen crosslinking therapy; however, 10-20% of these patients need corneal transplantation [28, 29]. KC is a sporadic disease, but genetic factors were still found [30]. Genetic variations in antioxidant defense genes such as CAT and GPX can reduce antioxidant capacity or increase OS, altering the risk of KC [31]. In addition, KC has been reported to be the result of genetic and environmental factors in which OS is involved. In KC patients, CAT RNA and activity and the ratio of lactic acid to pyruvate [32] in the cornea were increased, while arginine and the glutathione/oxidized glutathione ratio were reduced [33]. An increase in oxidant status was also reported in the sera of KC patients [34]. Meanwhile, the accumulation of MDA, peroxide, and hydroxyl free radicals and a reduction in antioxidant defense levels also suggest that patients with KC are exposed to strong OS, shifting the redox balance toward oxidation [35, 36]. A study found that HO-2 enzyme levels were lower in KC corneal epithelial cells. Liu and Yan found that KC increased ROS and increased keratometry and decreased the central cornea thickness. These changes were neutralized or reversed by sulforaphane (SFN) treatment through the Nrf2/HO-1 pathway [37].

#### 2.1.2. Fuchs' Endothelial Corneal Dystrophy (FECD)

FCED, an age-related cause of blindness with symptoms including poor vision, blurry cornea, poor night vision, and painful blinking, can eventually lead to full-layer corneal edema [38]. Corneal transplantation is the only way to restore vision loss in FECD patients. The main risk factors of FECD are family history, age (over 40), female sex, and smoking [39, 40]. FECD is a complex multifactorial inheritance disease with a variable expression rate and incomplete penetrance [41]. FECD-related genes include TCF4, COL8A2, ZEB1, AGBL1, SLC4A11, DMPK, LOXHD1, LAMC1, ATP1B1, and KANK4 [42–44], and environmental factors (especially UVA) also play an important role [45]. Due to its function and anatomical location, the corneal endothelial layer suffers from UVA exposure every day. The results of OS induced by UV usually include gene mutations, channelopathy, endoplasmic reticulum stress (ERS), and mitochondrial dysfunction [46]. The main characteristic changes due to FECD are collagen deposition in the Descemet membrane [47], cell morphological changes, apoptosis, and endothelial cell loss. Decreased antioxidant levels [48], deoxyribonucleic acid (DNA) damage, and apoptosis [49]; increased lipid peroxidation; excessive expression of cell senescence markers; and abnormal mitochondrial dynamics changes (decreasing mitochondrial DNA copy number, fragmented mitochondria, and increasing mitochondrial DNA damage) have been found in FECD [50]. Researchers found that Nrf2-mediated antioxidant defense and P53 play a key role in regulating FECD OS-induced apoptosis [49]. Cytoplasmic stability and the final translocation of Nrf2 are controlled by one of its stabilizers, DJ-1, which is decreased dramatically in FECD corneal endothelial cells (CECs) [51], accompanied by a decrease in Nrf2 and HO-1 [49] and impaired Nrf2 nuclear translocation [51]. These changes were attenuated to some extent by SFN, a Nrf2 activator. In both FECD CECs and an in vitro CEC OS model, SFN enhanced the nuclear transmigration of Nrf2, followed by the increasing expression of HO-1 and NQO1, decreasing expression of P53, and cell apoptosis reduction [52].

#### 2.1.3. Pterygium

Pterygium is a kind of vascularized connective tissue from the conjunctiva that invades the cornea from the side of the nose, presenting an inflammatory, proliferative, and invasive growth. Excessive exposure to UVB (especially by way of OS) is one of the most important causes of pterygium [35]. UVB radiation may cause toxic light damage to DNA directly or by increasing ROS, which causes DNA damage [53]. In patients with pterygium, the serum total oxidant status (TOS), total antioxidant status (TAS), and nitric oxide (NO) and MDA content were significantly increased, and the antioxidant enzyme (SOD, CAT, and GPX) content was decreased [54, 55], indicating an increase in nonenzyme antioxidant activity. Meanwhile, the DNA damage parameters tail length (TL), tail intensity (TI), and tail moment were significantly increased [56]. Immunohistochemical staining for 8-OHDG in the nucleus showed more extensive and deeper staining than that observed in the normal conjunctiva. In ELISA, the average amount of 8-OHDG in the pterygium tissue was 4.7 times greater than that in the normal conjunctival tissue [57], while the p53 protein level changed and inflammatory mediators were increased [58]. Proteasome beta 5 (PSMB5), which can degrade many aberrant and denatured intracellular proteins as well as functional proteins, is mediated by the Nrf2-ARE pathway in many cell types. Recent research has found that in conjunctival fibroblasts, Nrf2/ARE mediated the downregulation of PSMB5 and that these changes could be reversed by the Nrf2 activator oltipraz [59].

#### 2.1.4. Dry Eye

Dry eye, a multifactorial disease characterized by a loss of homeostasis of the tear film, appears together with ocular symptoms, ocular surface inflammation, and damage, and neurosensory abnormalities play etiological roles [60]. In recent years, air pollution has become increasingly serious (especially with the increase in fine particulate matter (PM2.5) and smoke) and has begun to be a factor that affects many diseases, including respiratory diseases [61] and hematological diseases [62]. Elementary carbon in PM2.5 produces a high concentration of ROS through the phagocytosis of macrophages, while organic carbon also produces ROS during its metabolism [63]. PM2.5 can inhibit SOD1 by promoting the expression of miRNA-206, leading to an increase in ROS and aggravating the pneumonia response and asthma symptoms [64]. Climate has also been shown to be associated with ocular surface integrity and tear film stability [65–67]. The tear film protects the ocular surface from many physical factors. Many antioxidants in the tear film, such as ascorbic acid, tyrosine, reduced glutathione, cysteine, and uric acid [68], serve as regulators in wound healing, corneal inflammatory response [69], and improving tear film stability. A study showed that the prevalence of dry eye is highest in the northern region of China, and it is lowest in the central region, suggesting that air pollution is associated with the onset of dry eye in which air pollutants including O_3_, PM2.5, and SO_2_ are potential risk factors [70]. The antioxidant enzymes SOD, CAT, and GPX were significantly less expressed in dry eye patients than in controls [71]. Furthermore, the expression of the lipid peroxidation markers 4-HNE and MDA was increased in dry eye patients with Sjøgren's syndrome and was closely related to tear film break-up time (BUT), Schirmer's tear value, tear clearance rate, keratoparaneliopathy score, conjunctival goblet cell density, and symptom score [72]. Some literature suggests that intervention with dietary supplements, vitamins, or omega-3 fatty acids can reduce OS [73]. Kojima et al. proposed that after exposure to sidestream cigarette smoke, Nrf2 KO mice had a significantly shortened BUT, significantly increased vital staining score, and reduced mucin 1 and Muc5ac staining compared to wild-type mice, suggesting that Nrf2 plays an important role in protecting eye surfaces from smoke exposure [74].

### 2.2. Cataract

Cataract is one of the most important causes of blindness worldwide, and age-related cataract is the most common type. Aging and OS are the main risk factors. Despite long-term exposure to UV, the lens has a well-established antioxidant system to combat OS and is rich in glutathione (GSH) [75]. Researchers used Lens Glutathione Synthesis KnockOut (LEGSKO) mice to develop a GSH defect model and confirmed that Nrf2 was the main upstream regulator of proteomic responses in LEGSKO lens fibroblasts [76]. However, with increasing age, GSH levels gradually decrease, and lens protein aggregation, DNA damage, lipid peroxidation, calcium homeostasis imbalance, and hydration occur, thus increasing lens turbidity [2, 77, 78]. In diabetic patients, the level of superoxide in the mitochondria is increased [79], and increased glucose utilization, insulin resistance, and OS lead to an increase in advanced glycation end products (AGEs) [80], which accelerates the formation of diabetic cataract.

As Nrf2 is a major antioxidant component, Nrf2 pathways regulate the expression of over 600 downstream antioxidant genes; imbalances in Nrf2 pathways have long been reported to be involved in the generation and development of cataract. With increasing age, the expression of Keap1 increases and that of Nrf2 decreases. Additionally, an increase in ROS also inhibits the antioxidant protective function of Nrf2 [81], which limits the transcription of downstream antioxidant enzymes, leading to a failure of the antioxidant system and accelerating the formation of cataract [82]. The protective mechanism of the ERS/unfolded protein response (UPR) is activated during the formation of most cataracts. Nrf2 pathways are activated under ERS to enhance the expression of multiple cellular protective enzymes that restore redox homeostasis [83]. The lens epithelial cells (LECs) of Nrf2 KO mice showed a higher cell death rate than those of wild-type mice treated with methylglyoxal at different concentrations. The above results indicated that normal Nrf2 levels are critical for lens survival under stress conditions [84].

Therefore, we have summarized some studies on the protection of LECs under stress through Nrf2 pathways. The use of Nrf2 activators (such as SFN pretreatment [85]) or the overexpression of Nrf2 [86] can reduce DNA fracture; upregulate Nrf2, NQO1, HO-1, etc. [87]; and protect LECs from OS damage. Puerarin [88], *Rosa laevigata* Michx. extract [89], hyperoside [90], acetyl-L-carnitine [91], morin [92], trimetazidine [93], rosmarinic acid [94], and DL-3-n-butylphthalide [95] have been shown to protect LECs from OS by activating the Nrf2 pathways. Besides, the inhibition of miRNA-4532 protected human LECs from UV-induced oxidative injury via activating SIRT6-Nrf2 signaling [96].

### 2.3. Glaucoma

Glaucoma is the second leading cause of irreversible human blindness worldwide, especially in elderly individuals, closely related to OS. Primary open-angle glaucoma (POAG) patients are susceptible to oxidative damage because their total reactive antioxidant capacity is reduced by 60%-70% [97]. Increasing evidence through clinical and experimental studies over the past decade has revealed that OS-induced dysfunction of trabecular meshwork cells (TMCs) can obstruct the outflow of the aqueous humor (AH), causing pathologically high intraocular pressure (IOP), which is consistent with the mechanical theory of glaucoma [98]. Pathologically high IOP can then cause retinal ganglion cell (RGC) mitochondrial dysfunction and apoptosis, therefore contributing to glaucoma vision loss [99].

The trabecular meshwork (TM) sustains OS due to the effects of the UV-ray-based oxidative byproducts of aqueous epithelial cells and CECs [100] and an imbalance between oxidants and antioxidants or excessive ROS accumulation [101]. TMCs under OS present typical changes observed in POAG, including extracellular matrix (ECM) accumulation, cell apoptosis, cell death, changes in the structure and function of the cytoplasm as well as lysosomes [102], and cytoskeletal disruption [103]. Cheng et al. found that Nrf2 expression was downregulated in glaucomatous TMCs compared to human TMCs and that in both cell types, the overexpression of Nrf2 could promote viability and reduce apoptosis [104].

Chronic hypertensive glaucoma and retinal ischemia caused by a sharp increase in IOP stimulate the production of ROS and dysregulate basic autophagy. The longer the injury lasts or the more dramatically increased the IOP is, the greater the extent of the immediate increase in autophagy is, inducing RGC death in a relatively short period of time [105]. Additionally, various proteins involved in cellular redox homeostasis and the OS response were upregulated in the retinas of ocular hypertensive humans [106]. In a retinal ischemia-reperfusion (I/R) model, the loss of neurons in the RGC layer was more severe in Nrf2 KO mice than in wild-type mice, and the RGC activity of Nrf2 KO mice was reduced, indicating that Nrf2 had an inherent protective effect in RGCs [107]. Repeated mild reperfusion led to chronic OS, especially in the mitochondria [108]. Virus-mediated delivery of Nrf2 effectively protected RGCs from oxidative damage after acute nerve damage [109].

Trabeculectomy, a classic glaucoma filtration surgery, destroys the structure of the conjunctiva and subconjunctival tissue and activates the immune system and the release of inflammatory cytokines. The activation of the vascular endothelial growth factor (VEGF-*α*) and the transforming growth factor (TGF-*β*) [110] leads to cell proliferation, migration, extracellular ECM formation, and collagen contraction, which promotes scar formation and is the key factor for surgical failure [111]. TGF-*β*1 causes cell apoptosis [112], fibrotic gene expression, and myofibroblast differentiation [113] due to ROS production and also inhibits the glutathione antioxidant system [114]. The miRNA-29 family is closely associated with TGF-*β*-mediated fibrosis [115, 116]. In patients with glaucoma, TGF-*β*2 was found to stimulate Tenon's capsule fibroblast proliferation via suppression of miR-29b expression regulated by Nrf2 [117], which indicates that Nrf2 may protect cells against TGF-*β* and even fibrosis by upregulating miR-29b.

### 2.4. Uveitis

Uveitis is a group of blindness-inducing autoimmune diseases. The mechanism of uveitis is not fully understood, but the imbalance between CD4+ CD25+ forkhead box protein+ T regulatory (Treg) cells and T helper 17 (Th17) cells is thought to be involved in the pathogenesis of autoimmune uveitis. The Nrf2 regulatory enzyme has been extensively studied in experimental autoimmune models because it plays an essential role in chemical reactions and provides a protective mechanism against autoimmune venereal diseases. In an encephalomyelitis mouse model, the absence of Nrf2 aggravated disease severity, which was reduced by treatment with SFN [118] or downregulation of the negative Nrf2 regulator Keap1 [119]. Nrf2 inhibited suppressive helper 1 (Th1) and Th17 cell responses and activated immunosuppressive Treg and Th2 cells [120], thus exerting protective effects.

In 2009, Nagai et al. confirmed the hypothesis that the Nrf2 pathway protects against injury in experimental uveitis by attenuating OS and modulating the innate immune response [121]. Chen et al. that found sodium butyrate (NaB) had great potential for inducing Treg cells in an experimental uveitis model. In vivo, NaB treatment reduced the number of Th17 cells in the spleens of mice with autoimmune uveitis and increased the number of Treg cells. In vitro, NaB treatment transformed original T cells from Th17 cells to Treg cells, and the inhibition of Th17 differentiation and the protective effect of NaB on uveitis may have been achieved by the Nrf2/HO-1 pathway [122].

### 2.5. Retinopathies

The anterior segment of the eye absorbs more than 99% of UV radiation, but the other 1%, especially UVA radiation, reaches the retina [123], leaving the retina continuously exposed to ROS and causing OS. OS is the main factor of retinal degeneration related to aging, such as age-related macular degeneration (AMD), and has also been linked to retinal inflammation and neuron degeneration. Furthermore, retinal I/R injury has been associated with the mechanisms of retinal vascular occlusion (RVO) and diabetic retinopathy.

#### 2.5.1. Diabetic Retinopathy (DR)

DR is a common and progressive diabetic complication and the leading cause of blindness in the diabetic population. Historically, DR was described as an ocular microvascular disease caused by metabolic disorders (especially elevated glucose levels, oxidative phosphorylation, increased AGEs, and aldose reductase activity), increased ROS, and mitochondrial dysfunction, causing OS in only retinal cells and capillaries [124]. New evidence suggests that diabetes causes considerable damage to retinal neurons in the early stage of the disease [125, 126]. In the diabetic retina, neuronal apoptosis and the activation of neurogliocytes may also cause OS, thus creating a vicious cycle.

Nrf2 significantly contributes to protecting diabetic retinal cells from OS damage and inhibiting vascular inflammation. In animal experiments, Nrf2 KO mice showed significantly increased superoxide levels, glutathione was reduced, and early blood-retinal barrier dysfunction and the onset of neuron dysfunction were observed [127]. As for the anti-inflammatory protective effect of Nrf2, Nrf2 KO mice showed increased inflammation factors [128]. In endothelial cells exposed to high glucose or in the DR retinas, damage was observed which was prevented by the Nrf2 inducer t-BHQ and small interfering RNA (siRNA) against Keap1 [129]. C1q/TNF-related protein 3 (CTRP3) plays a role in the progression of diabetes and its complications, whose overexpression improved cell viability of high-glucose- (HG-) induced retinal pigment epithelium (RPE) cells by the activation of the Nrf2/HO-1 pathway [130]. In another study, the upregulation of casein kinase 2 interacting protein-1 (CKIP-1) inhibited HG-induced inflammation and OS in human retinal endothelial cells (RECs) and attenuated DR by modulating the Nrf2/ARE signaling pathway [131]. Forkhead box class O6 (FOXO6) is a member of the FOXO family that can regulate diabetes-induced OS, and the suppression of FOXO6 protects ARPE-19 cells from HG-induced OS and apoptosis, which is in part mediated by the activation of the Akt/Nrf2 pathway [132]. Activin receptor-like kinase 7 (ALK7), one of type I transforming growth factor *β* receptors, is involved in metabolic regulation, whose knockdown results in an increase in the expressions of Nrf2 and HO-1 in ARPE-19 cells in response to HG induction [132].

Recently, an increasing number of researchers have used Nrf2 inducers or activating agents, such as RS9 [133], CDDO-Im [126], dh404 [134], CKIP-1 [131], CTRP3 [130], curcumin [135], and SFN [136], to study the protective effect of Nrf2 against DR injury and made significant progress (see Table 1).

#### 2.5.2. Age-Related Macular Degeneration

*Age-related macular degeneration* (*AMD*), a kind of eye disease, mainly affects elderly people for which aging is the most severe risk factor. Complement factor H (CFH) mutation is a visible part of the genetic changes in AMD patients [137]. In addition to OS, there are environmental/lifestyle factors, including smoking, obesity, and a high-fat or low-antioxidant diet, and environmental factors such as exposure to UV radiation and blue light [138]. OS occurs in the above conditions and plays a vital role in the pathogenesis of AMD. AMD is associated with the progressive degeneration and death of RPEs, followed by adverse effects on rods and cones [139]. The ROS level was shown to be significantly higher in the RPEs of AMD patients than in those of a control group [140, 141]. RPEs are chronically affected by OS due to their exposure to the outer layer of photoreceptors, leading to a decrease in antioxidant enzyme levels [142] and an increase in the OS products MDA and 4-HNE, AGEs, and oxidation-specific epitopes in the macular area [143]. OS also accounts for the reduced number of mitochondria, lower mitochondrial matrix density, and mitochondrial DNA (mtDNA) mutation [144]. For the above reasons, researchers have performed much research on the antioxidant process in AMD, the most gratifying of which is that on the participation of Nrf2.

Aging can reduce Nrf2 mRNA or protein levels, thus weakening Nrf2 signal transduction. The retinas of Nrf2 KO mice [145] and si-Nrf2-transfected RPEs [146] were more susceptible to OS, which accelerated photoreceptor cell death; this damage could be alleviated by amplifying the endogenous Nrf2 pathway with electrophilic drugs or locally targeted antioxidant drugs [145, 147].

#### 2.5.3. Retinitis Pigmentosa

Retinitis pigmentosa (RP) is a group of inherited retinal diseases characterized by rod and cone photoreceptor degeneration [148, 149]. As the rod cell pole accounts for approximately 95% of the total number of photoreceptors, it consumes most of the oxygen delivered to the retina; when a variety of genetic mutations cause rod cell death, the cone oxygen load increases and cone cells then die gradually [150, 151], eventually leading to tubular vision and blindness. A reduced GSH/GSSG ratio is a marker of OS in tissues. In patients with RP, the ratio of GSH/GSSG in the aqueous humor was significantly reduced, and the total antioxidant capacity and SOD3 activity and protein concentration were decreased [152], while the water-based protein carbonyl content was significantly increased. Also, the decreased activity of SOD3 in the peripheral blood, the increased formation of thiobarbituric acid-reactive substances, and the upregulated nitric oxide/ring GMP pathway were observed. These findings support the hypothesis that the antioxidant capacity of the eye is reduced in patients with RP [153].

Since Nrf2 is important for OS regulation, is Nrf2 involved in RP? The answer is yes. In 2009, Usui et al. proposed that increased expression of catalase and SOD 2 could reduce cone cell death in RP [154]. Xiong et al. delivered SOD2, CAT, and Nrf2 to the cones of an RP mouse model using adenoassociated virus carriers and found that the overexpression of Nrf2 was the most effective in saving cells, preserving the survival of RGCs after nerve compression and improving retinal morphology [109]. Wang et al. proposed that the absence of the Sigma 1 receptor (Sig1R) accelerated the death of photoreceptor cells in RP mice [155], and in their subsequent experiments, they concluded the protection of cones mediated by Sig1R required Nrf2 [156]. In a mouse model of retinal degeneration treated with SFN, significantly higher electroretinographic (ERG) a- and b-wave amplitudes and decreased photoreceptor cell death were observed [157]. These studies all provide a theoretical basis for the therapeutic potential of Nrf2 in RP.

### 2.6. Optic Neuritis

Optic neuritis, a disease defined by autoimmune demyelination of the optic nerve and the death of RGCs, is associated with visual impairment and multiple sclerosis (MS). In laboratory studies of optic neuritis, an experimental autoimmune encephalomyelitis (EAE) model of recurrent sclerosis is often used as a model of optic neuritis [158]. In 1998, Guy et al. tested the inhibition of oxidative damage in the optic nerves of EAE animals through virus-mediated CAT gene transfer and found that H_2_O_2_-related enzyme gene expression could decrease demyelination by 38%, swelling of the optic nerve head by 29%, and lymphocytes by 34% compared with the control [159]. Qi et al. also concluded in 2007 that with the inhibiting SOD2 expression, myelin fiber injury was increased by 27%. With the overexpression of the SOD2 level, myelin fiber damage was reduced by 51% and the RGC loss was saved by a factor of four [160]. When the role of Nrf2 was examined, Nrf2 KO EAE mice showed more severe visual impairment, optic nerve inflammation, and RGC degeneration, indicating that Nrf2 had a neuroprotective effect in EAE-related optic neuritis. The overexpression of Nrf2 increased RGC survival in an EAE model of optic neuritis [161]. Dimethyl fumarate (DMF) has been used in the treatment of MS [162], experimental Parkinson's diseases [163], long-term memory deficits [164], and other diseases of the nervous system. Recently, DMF was confirmed to reduce the severity of optic neuritis and retain vision and RGCs by the Nrf2 pathways. We believe these findings provide a useful perspective for the treatment of optic neuritis [165].

## 3. Novel Strategies for Activating Nrf2

### 3.1. Noncoding RNAs

MicroRNAs are a class of small noncoding RNAs (19–25 nucleotides) that regulate a wide range of cellular processes by repressing the transcription or translation of their target genes. lncRNAs are >200-nucleotide-long RNA molecules that lack or have limited protein-coding potential but can regulate transcription in *cis* or *trans*, the organization of nuclear domains, and RNA or protein formation through several different mechanisms [166, 167]. Recently, noncoding RNAs became a hot topic in scientific research, even in ocular research, as shown in Table 2. Noncoding RNAs provide an attractive opportunity to defend against OS for the diagnosis and prognosis of ocular diseases.

### 3.2. Exogenous Compounds

Many types of compounds have anti-inflammatory, antioxidant, and antifibrotic properties by directly targeting Nrf2 and Nrf2 repressors (Keap1, Bach1, and c-Myc) [168–170] and have potential preventative functions in eye diseases. Interest in investigating exogenous compounds has revealed new treatment options against OS. Curcuminoids, cinnamic acid derivatives, coumarins, chalcones, flavonoids, and terpenoids are typical Nrf2 inducers. In addition, phenols and quinones (e.g., t-BHQ) [171], polyphenolic flavonoids (e.g., quercetin) [172], stilbenoid and nonflavonoid polyphenolic compounds (e.g., resveratrol) [173], and other compounds including SFN, sphaeropsidin A (SA), CDDO-Im, long-acting (1R)-isopropyloxygenipin (IPRG001), omaveloxolone, astaxanthin [174], and lycopene [175] activate Nrf2 and upregulate some downstream Nrf2 genes.  Table 1 lists studies on Nrf2 activators connected to ocular diseases.

## 4. Nrf2: Negative Side

Although Nrf2 has many antistress functions, scientists have also revealed the dark side of Nrf2, which might be a driver role of cancer progression [176]. There are cancer-associated mutations that activate Nrf2 [177, 178]. Also, when ROS exceeds the critical threshold, Nrf2 binds to the ARE gene of Klf9 and upregulates Klf9 expression. Klf9, in turn, inhibits Trx reductase two expression, amplifying the ROS cascade and ultimately leading to cell death [179]. PI3K/AKT signaling and Nrf2 signaling are increased in cells with mutant PTEN, resulting in higher proliferation rates and increased tumorigenicity [180]. Therefore, it is essential to determine the boundary between the beneficial and potentially harmful effects of Nrf2 activation. Ongoing clinical trials will undoubtedly provide important progress in answering these questions in the coming years.

## 5. Conclusion and Prospect

More and more evidence show that Nrf2 plays a certain role in the occurrence and development of ocular diseases. Therefore, Nrf2 might be a potential target of protecting ocular cells from various stresses and preventing ocular diseases. We are looking forward to come across more clinical studies.

## Figures and Tables

**Table 1:** Nrf2 regulative function of exogenous compounds.

Name	Experimental subjects	Functions and mechanisms
Edaravone	CECs	Augments the expression of Nrf2 and its target genes, such as HO-1, GPX-1, and GCLC [23]
SERPINA3K (SA3K)	CECs	Protects against oxidative stress by targeting the ROS generation/degradation system and modulating the Keap1-Nrf2 signaling pathway [189]
	Cultured pterygial epithelial cells (PECs)	Inhibits NADPH oxidase 4 (NOX4) and elevates Nrf2, NQO1, and SOD2 [190]
Rosmarinic acid (RA)	PECs	Reduces the cell viability of PECs. Increases Nrf2, HO-1, and NQO1 expression and activates SOD and CAT [191]
Chondrocyte-derived extracellular matrix (CDECM)	Human conjunctival epithelial cells (ConECs) and PECs	Inhibits NF-*κ*B activation and improves Nrf2 induction by blocking the p38 MAPK and PKC signaling pathways [192]
4-HNE	CECs	Elevates the ROS generation enzyme NOX4 and induces Nrf2 and its downstream effectors [193]
	RPEs	Increases Nrf2 activity and GSH synthesis in a dose-dependent manner [194]
Trichostatin A (TSA)	Corneal fibroblasts	Inhibits TGF-*β*-induced ROS accumulation and myofibroblast differentiation via enhanced Nrf2-ARE signaling [195]
DL-3-n-butylphthalide (NBP)	Rat diabetic cataract model	Delays the onset and progression of diabetic cataract by enhancing the expressions of Nrf2, TRX, and CAT [95]
Acetyl-L-carnitine	LECs	Prevents homocysteine-induced suppression of Nrf2/Keap1-mediated antioxidation [91]
Puerarin	Rat diabetic cataract model	Prevents cataract development and progression in diabetic rats through Nrf2/HO-1 signaling [88]
	RPEs	Activates Nrf2/HO-1 antioxidant signaling pathway [196]
Rosmarinic acid	Sprague-Dawley rat pup selenite-inducedcataractogenesis model	Increases the protein expressions of filensin, calpain 2, Nrf2, SOD, HO-1, and NQO1; the antioxidant enzyme activities; and the GSH level [94]
Morin	LECs	Induces HO-1 via the ERK-Nrf2 signaling pathway [92]
Calcium dobesilate	Rat D-galactose-induced cataract model	Increases Nrf2 and HO-1 levels and inhibits the Keap1 level [197]
*Rosa laevigata* Michx.	LECs	Inhibits ROS production and elevates mitochondrial membrane potential, through the induction of HO-1 expression mediated by the PI3K/Akt and Nrf2/ARE pathways [89]
Hyperoside	LECs	Increased Nrf2 level and the binding activity of its antioxidant components, increased the expression of HO-1, and restored cell vitality through ERK [90]
Quercetin	TMCs	Upregulates antioxidant peroxiredoxins through the activation of the Nrf2/Nrf1 transcription pathway [198]
	RPEs	Protects RPEs from H_2_O_2_-induced cytotoxicity by activating the Nrf2 pathway [199]
Sodium butyrate	Mouse experimental autoimmune uveitis (EAU) model	Regulates Th17/Treg cell balance to ameliorate uveitis via the Nrf2/HO-1 pathway [122]
Lutein	RPEs	Reverses hyperglycemia-mediated blockage of Nrf2 translocation by modulating the activation of intracellular protein kinases [200]
Dh404	Müller cells	Reduces Müller cell gliosis and vascular leakage as well as the hypoxia-induced increase in ROS and angiogenic factors with a concomitant increase in Nrf2-responsive antioxidants [134, 201]
Myricetin derivatives (F2)	RPEs	Protects RPE cells against OS by the activation of Nrf2 and SOD2 [202]
Fenofibrate	Mice diabetic retinas	Increases the expression of Nrf2, NQO1, and HO-1 [203]
Probucol	Müller cells	Activates the Keap1/Nrf2/ARE pathway [204]
Homocysteine	Müller cells	Increases the expression of Nrf2, NQO1, and HO-1 [205]
Ebselen	Müller cells	Reduces the ROS levels and increases the expression of Nrf2, HO-1, glutathione peroxidase-1, NQO1, and glutamate-cysteine ligase [206]
DL-3-n-butylphthalide	Müller cells	Increases the expression level of HO-1 in a time-dependent manner [207]
3H-1,2-dithiole-3-thione	RPEs	Induces Nrf2 phosphorylation, causing Nrf2 disassociation with Keap1 and its subsequent nuclear accumulation [208]
Curcumin	RPEs	Prevents high glucose damage through ERK1/2-mediated activation of the Nrf2/HO-1 pathway [135]
Glycyrrhizin	RPEs	Protects against sodium iodate-induced RPE and retinal injury through activation of the Akt and Nrf2/HO-1 pathway [209]
Escin	RPEs	Activates Akt-Nrf2 signaling [210]
Ginsenoside Rh3	RPEs and mice retinas	Rh3 induces miRNA-141 expression causing the downregulation of Keap1 and activating Nrf2 [211]
Hesperetin	RPEs	Upregulates the Keap1-Nrf2/HO-1 signal pathway [212]
Lycopene	RPEs	Inhibits ICAM-1 expression and NF-*κ*B activation by Nrf2-regulated cell redox state [213]
4-Acetoxyphenol	RPEs	Blocks the increase of cellular ROS and upregulates NQO1 and HO-1 genes by stabilizing and inducing the nuclear translocation of Nrf2 [214]
Taxifolin	RPEs	Enhances the nuclear accumulation of Nrf2 and increases the expression of HO-1, GCLC, GCLM, and NQO1 [215]
Genipin	RPEs	Reverses the inhibitory effects of H_2_O_2_ by promoting cell viability, attenuating ROS accumulation and cell apoptosis, and increasing the expression of Nrf2, HO-1, and NQO1 [216]
*α*-Tocopherol	RPEs	Activates the Keap1/Nrf2 pathway by increasing Nrf2 expression and inducing its translocation to the nucleus, and increases HO-1 as well as NQO1 [217]
Astaxanthin	RPEs	Activates the Nrf2-ARE pathway by inducing Nrf2 nuclear localization and increasing NQO1, HO-1, GCLM, and GCLC [218]
Salvianolic acid A	RPEs	Causes Nrf2 phosphorylation, accumulation, and nuclear translocation and increases the expression of HO-1 [219]
Salvianolic acid B	RPEs	Protects cells from OS-induced cell death by activating glutaredoxin 1 [220]
Lipoamide	RPEs	Induces the expression of Nrf2 and its translocation to the nucleus, leading to an increase in the expression or activity of NQO1, GST, GCL, catalase, and Cu/Zn SOD [221]
Thymoquinone	RPEs	Enhances the activation of the Nrf2/HO-1 signaling pathway [222]
Blueberry anthocyanins	Diabetes rat retinas	Increases the mRNA levels of Nrf2 and HO-1, and the nuclear location of Nrf2 and protein levels of HO-1 [223]
Grape seed proanthocyanidin extract	Diabetes rat retinas	Increases the expression of Nrf2 and HO-1 [224]
Carbamyl erythropoietin	Diabetes rat retinas	Increases the expression of Nrf2, HO-1, and NQO1 [225]
Ginsenoside Rb1	Diabetes rat retinas	Increases the expression of Nrf2, GCLC, and GCLM [226]
Pyridoxamine	Retinal photoreceptor cells	Partly protects cells against light damage by Nrf2 expression [227]
Scutellarin (SC)	RECs and RPEs	Enhances nuclear Nrf2 accumulation and the SC-provided alleviation on BRB breakdown in STZ-induced diabetic mice was diminished in Nrf2 knockout mice [228]
Delphinidin (2-(3,4,5-trihydroxyphenyl) chromenylium-3,5,7-triol)	RPEs	Reverses the decreased activities of SOD, CAT, and GSH-PX and the elevated MDA level via increasing nuclear Nrf2 protein expression [229]
4-Hydroxy-7-oxo-5-heptanoate (HOHA) lactone	RPEs	Induces upregulation of Nrf2, GCLM, HO-1, and NQO1 [230]
Melatonin	The retina of diabetic rats	Significantly upregulates glutamate-cysteine ligase by retaining Nrf2 in the nucleus and stimulating Akt phosphorylation [231]
Galangin	RECs and RPEs	Alleviates DR by reversing TNF*α*-induced blood-retinal barrier dysfunction by abrogating oxidative stress injury via activating Nrf2 [232]
Nonsteroidal anti-inflammatory drugs (NSAIDs)	Laser-induced CNV model in rat; RPEs	Inhibits neovascularization of choroid through the HO-1-dependent pathway [233]
RS9	Retinal microvascular endothelial cells (RMECs)	Decreases retinal neovascularization through suppressing VEGF expression and increasing Nrf2, HO-1, and NQO1 [234]
	661W cells and Müller glia cells	Enhances the expression of Nrf2 and increases the expression of HO-1, GCLC, GCLM, and NQO1 [235]
Acetaldehyde dehydrogenase 2 (ALDH2)	Diabetic rats' retinas	Increases the expression of Nrf2 [236]
Lipoic acid	RGCs	Induces the expression of HO-1 by promoting the translocation of Nrf2 to the nucleus [237, 238]
Sulforaphane	FECD corneal specimens	Enhances cell viability by decreasing ROS. Enhances nuclear translocation of Nrf2, DJ-1, HO-1, and NQO1 and decreases p53 [52]
	Müller cells	Enhances the nuclear accumulation of Nrf2 and increases the expression of HO-1 and NQO1 [136]
	Rabbit KC corneas	Protects corneas against oxidative stress injury through activation of the Nrf2/HO-1 antioxidant pathway [37]
	LECs	Includes the expression of NQO1 and TXNRD1 and the Nrf2 translocation to the nucleus [87, 239]
	TMCs RGCs	Attenuates H_2_O_2_-induced oxidative stress via PI3K/Akt-mediated Nrf2 signaling activation [240, 241]
CDDO-Im	661W	Inhibits ROS, increases OS, and increases neuronal cell survival after I/R injury [107]
	Nrf2 KO mice	Increases expressions of Nrf2, HO-1, NQO1, and GCLM in the retina, reduces inflammatory mediator expression, and reduces leukocyte adherence to retinal vasculature [121]
RTA 408	RPEs	Activates Nrf2 and increases the expression of its downstream genes, such as HO-1, NQO1, SOD2, catalase, Grx1, and Trx1 [242]
Soluble P-selectin	RGCs	Increases NQO1 and HO-1 expression levels, along with increased transcription factor Nrf2 [243]
Eriodictyol	RGCs	Enhances the nuclear translocation of Nrf2 and elevates the expression of HO-1 [244]
	RPEs	Induces the nuclear translocation of Nrf2, enhances the expression of HO-1 and NQO1, and increases the levels of intracellular glutathione [245]
Neuroligin-3	RGCs and RPEs	Activates Nrf2 signaling and enables Nrf2 protein stabilization, nuclear translocation, and expression of HO-1, NOQ1, and GCLC [246]
Long-acting (1R)-isopropyloxygenipin (IPRG001)	RGCs	The protective action depends on NO induction and the Nrf2/HO-1 antioxidant response element pathway by S-nitrosylation [247]
Resveratrol	RGCs	Upregulates the expression of Nrf2, HO-1, and NQO1 [248, 249]
L-carnitine (LC)	RGCs	Increases levels of Nrf2, HO-1, and *γ*-GCS and decreases expression of Keap1 protein [3]
SNJ-1945 (an exogenous calpain inhibitor)	RGCs	Protects RGCs against OS induced by high glucose [250]
Monomethyl fumarate	Ganglion cell layer	Fumaric acid esters exert a neuronal protective function in the retinal I/R model via Nrf2 modulation [251]
Trimetazidine	RGCs	Confers protection against RGC apoptosis via Nrf2/HO-1 signaling [252]
	LECs	Reduces ROS production, inhibits Keap1 demethylation, and rescues Nrf2 expression level [93]
Hydrogen sulfide gas (H_2_S) donor drugs	RGCs	Increases the levels of Nrf2 and HO-1 and inhibits OS-induced cell death [253]
5*α*-Androst-3*β*, 5*α*, 6*β*-triol (TRIOL)	RGCs	Activates and upregulates Nrf2 and HO-1 by negative regulation of Keap1 [254]
Nipradilol	RGCs	Protects RGCs through S-nitrosylation of Keap1 and HO-1 induction [255]
Flavonoids	RGCs	Induces Nrf2 and HO-1 [256]
	RPEs	Induces the expression of Nrf2 and HO-1 [257]
Sulbutiamine	RGCs	Stimulates CAT and significantly increased Nrf2 and HO-1 levels [258]
Chalcone analog L2H17	RGCs	Exhibits its antioxidative effects by activating the Nrf2 pathway [259]
Chlorogenic acid	RGCs	Relieves oxidative stress injury in retinal ganglion cells through lncRNA-TUG1/Nrf2 [260]

**Table 2:** Nrf2 regulative function of noncoding RNAs.

Name of miRNAs	Experimental subjects	Functions and mechanisms
miR-4532	Human lens epithelial cells	Inhibition of miR-4532 protects HLECs from UVR-induced oxidative injury via activation of the SIRT6-Nrf2 pathway [96]
miR-27a	TMCs	Regulates Nrf2 expression at the posttranscriptional level [181]
miR-29b	Tenon's capsule fibroblast	TGF-*β*2 fibroblast proliferation via suppression of miR-29b expression regulated by Nrf2 [117]
miR-93	TMCs	Inhibits viability and inducing apoptosis of the GTM cells by the suppression of Nrf2 [97, 182]
miR-141	RGCs	Attenuates UV-induced oxidative stress via activating Keap1-Nrf2 signaling [183]
miR-144	RPEs	Inhibiting microRNA-144 potentiates Nrf2-dependent antioxidant signaling and protects against OS-induced outer retinal degeneration [184]
miR-626	RPEs	Ectopic overexpression of miR-626 targeting the 3′-UTR (3′-untranslated region) of Keap1 downregulates its expression, promoting Nrf2 protein stabilization and nuclear translocation, leading to the expression of HO-1, NOQ1, and GCLC [185]
miR-601	RPEs	Overexpression of miR-601 inhibits Cul3 3′-UTR activity and downregulates Cul3 expression, leading to Nrf2 protein stabilization and its nuclear translocation as well as expression of HO-1, NQO1, and GCLC [186]
lncRNA-MEG3	Tenon's capsule fibroblast	The functional interaction between lncRNA-MEG3 and Nrf2 constitutes the mechanism by which TGF-*β*2 induces Tenon's capsule fibroblast proliferation after glaucoma filtration surgery via the direct binding of MEG3 to Nrf2 [187]
lncRNA-Sox2OT	RGCs	Sox2OT knockdown plays an antioxidative role via regulating Nrf2/HO-1 signaling activity [188]
